# Different modes of variation for each BG lineage suggest different functions

**DOI:** 10.1098/rsob.160188

**Published:** 2016-09-14

**Authors:** John Chattaway, R. Andrei Ramirez-Valdez, Paul E. Chappell, Joseph J. E. Caesar, Susan M. Lea, Jim Kaufman

**Affiliations:** 1 Department of Pathology, University of Cambridge, Cambridge CB2 1QP, UK; 2 Sir William Dunn School of Pathology, University of Oxford, Oxford OX1 3RE, UK; 3 Department of Veterinary Medicine, University of Cambridge, Cambridge CB3 0ES, UK

**Keywords:** avian, gene conversion, segmental exchange, selection, SKINT, B-G

## Abstract

Mammalian butyrophilins have various important functions, one for lipid binding but others as ligands for co-inhibition of αβ T cells or for stimulation of γδ T cells in the immune system. The chicken BG homologues are dimers, with extracellular immunoglobulin variable (V) domains joined by cysteines in the loop equivalent to complementarity-determining region 1 (CDR1). BG genes are found in three genomic locations: BG0 on chromosome 2, BG1 in the classical MHC (the BF-BL region) and many BG genes in the BG region just outside the MHC. Here, we show that BG0 is virtually monomorphic, suggesting housekeeping function(s) consonant with the ubiquitous tissue distribution. BG1 has allelic polymorphism but minimal sequence diversity, with the few polymorphic residues at the interface of the two V domains, suggesting that BG1 is recognized by receptors in a conserved fashion. Any phenotypic variation should be due to the intracellular region, with differential exon usage between alleles. BG genes in the BG region can generate diversity by exchange of sequence cassettes located in loops equivalent to CDR1 and CDR2, consonant with recognition of many ligands or antigens for immune defence. Unlike the mammalian butyrophilins, there are at least three modes by which BG genes evolve.

## 1. Background

Many members of the B7 gene superfamily are cell surface molecules involved in regulation of the immune response, but some have functions outside of the immune system [1]. Soon after the first mammalian B7 molecule was identified as important in immune co-stimulation, two other members were described with non-immune functions: myelin oligodendrocyte glycoprotein (MOG), which is found on the membranes sheathing neurons, and butyrophilin (now called Btn1A1), which is involved in the structure of milk fat globules [2–5]. Subsequently, the other butyrophilin (Btn), the Btn-like, and the selection and upkeep of intraepithelial T cells (SKINT) genes were described with important functions in the immune system. The Btn and Btn-like genes function as stimulatory or inhibitory co-regulators of αβ T cells, or as ligands for γδ T cells (reviewed in [6–9]). In humans, some Btn genes have striking genetic associations with a variety of diseases (reviewed in [6–9]). The prototypic SKINT gene is involved in thymic education of intraepithelial γδ T cells found in mouse skin, although the single human SKINT gene is a pseudogene [10,11]. In chickens, one authentic Btn gene (Tvc-1 or Btn1A1) was discovered as a cellular receptor for avian leukosis virus subtype C and later another one (Btn2A1) was predicted by bioinformatics in the major histocompatibility complex (MHC) [12,13]. Otherwise the most closely related are the BG genes (also referred to as B-G in the older literature), long suspected to have roles in the immune response (reviewed in [14]).

The defining feature of the B7 gene superfamily is the presence of at least one characteristic extracellular domain with similarities to immunoglobulin (Ig) variable (V) regions, but otherwise the genes vary enormously [1]. The BG genes encode a single V-like domain, followed by a transmembrane region and a long cytoplasmic tail composed mainly of seven amino acid repeats. BG molecules are dimers, with the V-like domains disulfide-linked through cysteines in the loop corresponding to complementarity-determining region 1 (CDR1) in antibodies and T cell receptors (TCRs), with the transmembrane regions flattened to interact, and with the cytoplasmic tails presumably making a long α-helical coiled coil [15–17]. The extracellular domains are known to be polymorphic, since the chicken MHC was discovered as the B serological blood group for which the antibodies were primarily against BG molecules [18].

**Figure 1.**
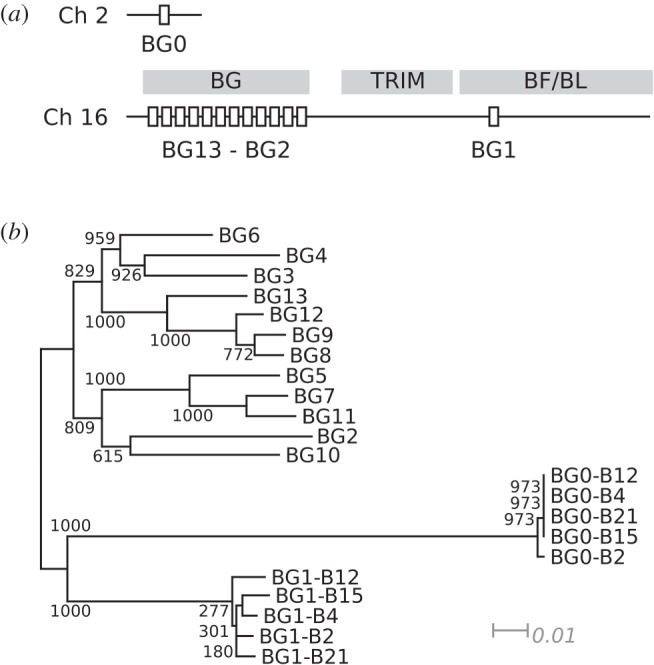
BG0, BG1 and the BG genes from the BG region occupy distinct genomic locations and form separate lineages. (*a*) Genomic locations of the BG genes. The BG, TRIM and BF/BL regions are indicated by grey boxes; only BG genes are depicted (as white boxes). Chromosome 2, Ch 2; chromosome 16, Ch 16. (*b*) NJ tree of the BG cDNA nucleotide sequences, excluding the cytoplasmic tail and non-orthologous bases at the extreme 5′ end. BG cDNA sequences are from the B12 haplotype unless otherwise stated. Genetic distance indicated; bootstrap values at the branch points.

Recent work [17] defining the genomic location and organization of BG genes shows that there are three locations of BG genes in chickens of the B12 MHC haplotype: the BG0 gene on chicken chromosome 2, the BG1 gene in the BF-BL region of the B locus (that is, the classical MHC) on chromosome 16, and a tandem array of 12 BG genes in the BG region nearby (figure 1*a*). The BG genes in the BG region show significant copy number variation (CNV) along with the presence of hybrid genes and other features associated with expansion and contraction of a multi-gene family by unequal crossing-over and deletion [17]. We wish to understand the evolutionary pressures upon the BG gene sequences in comparison to Btn and SKINT genes, and to begin to infer function of individual BG genes. Only limited information is available on BG sequence variation, and that only at the genomic level [17,19]. Here, we examine full-length cDNA for BG0 and BG1 from several haplotypes and several tissues, and also compare some closely related BG genes in the BG region to infer potential functional roles of BG molecules.

## 2. Material and methods

### 2.1. Materials

Experimental chicken lines from the Pirbright Institute (formerly the Institute for Animal Health) and maintained in Cambridge have been described [20,21], including line 6_1_ (MHC B2 haplotype), C (B4 and B12), 15I (B15) and 0 (B21).

### 2.2. Experimental methods

Total RNA was extracted from 30 mg tissue (spleen, thymus, bone marrow, duodenum and kidney) using the Nucleospin RNA II RNA extraction kit (MACHEREY-NAGEL), and cDNA was made using the RevertAid First Strand cDNA Synthesis Kit (Thermo Scientific), both as per manufacturer's instructions. The use of a heat-tolerant reverse transcriptase enzyme was found to be important for the creation of full-length BG cDNA. Amplification of near full-length BG cDNA was carried out with 20 U Phusion High-Fidelity DNA Polymerase (New England Biolabs) with 1 × HiFi buffer (containing 1.5 mM MgCl_2_), 40 µM each of dGTP, dATP, dCTP and dTTP, 0.4 mM of each primer and 1 µl cDNA in 25 µl total volume using a DNA Engine Tetrad 2 Peltier thermocycler PTC-0240G (BioRad) with reactions as follows: 95°C for 2 min, then 5 cycles of 95°C for 45 s, 60°C for 30 s, 72°C for 90 s, followed by 30 cycles of 95°C for 45 s, 60°C for 1 s, 72°C for 90 s, with a final 72°C extension step of 10 min at the end. The primers and additions used for BG0 were uc219 (GCACCACCAGAGAGGACAGCC) and uc220 (ACAATGGGATTAACACCCAAAGCAGT), and for BG1 were uc329 (GGCCACTCTCTTCTCCTACAGT) and uc333 (TGGGGTTGATACCCAAAGCAGTT) with an additional 0.5 mM MgCl_2_ added. Amplicons were gel purified using QIAquick Gel Extraction kit (Qiagen) according to manufacturer's instructions, concentrated by ethanol precipitation, cloned into pJET1.2 (Fermentas) according to manufacturer's instructions, and plated out on LB with 0.1 mg ml^−1^ ampicillin. Bacterial colonies were screened by colony PCR with 0.5 U recombinant TAQ (Invitrogen 10342020), 1 × Invitrogen PCR reaction buffer, 3 mM MgCl_2_, 0.25 µM each of dGTP, dATP, dCTP and dTTP, and 0.2 mM each primer: uc252 (CGACTCACTATAGGGAGAGCGGC) and uc458 (AAGAACATCGATTTTCCATGGCAG) in 20 µl total volume with reactions as follows: 95°C for 5 min, then 25 cycles of 94°C for 30 s, 60°C for 30 s, 72°C for 90 s, with a final 72°C extension step for 10 min. Minipreps were prepared using GenCatch Plasmid DNA Mini-Prep Kit (EPOCH Biolabs). Sanger sequencing using primers T7 (forward TAATACGACTCACTATAGGG), pJET1.2 reverse sequencing primer (AAGAACATCGATTTTCCATGGCAG) and two internal reverse primers uc453 (TGTKGTGYTGYGCTGCCA) and c345 (GGAGGAGGTGCAGAGCCACGAGATAAGGCAGGA) was carried out by the University of Cambridge Biochemistry Department sequencing service.

### 2.3. Analysis

Sequences were assembled using EMBOSS v. 6.1.0 tools trimseq, revseq, merger and descsec (http://packages.ubuntu.com/lucid/science/emboss) run inside bash v. 4.1.5 (www.gnu.org/software/bash) and perl v. 5.10.1 (www.perl.org) scripts, and analysed using CLC Workbench v. 5.7.1 (www.clcbio.com). Phylogenetic trees were constructed by neighbour joining (NJ) with 1000 bootstrap samples using ClustalX2 (www.clustal.org), then viewed in Dendroscope 3 (http://dendroscope.org). d*N*/d*S* values were calculated in MEGA6 (www.megasoftware.net) using the Nei and Gojobori method with p-distance parameter, overall average scope and 500 bootstrap replicates. Figures were made using Inkscape (https://inkscape.org). Synteny comparisons were performed with Ensembl release 78 (December 2014 at www.ensembl.ac.uk): human GRCh38, mouse GRCm38p3 and chicken Galgal4.

### 2.4. Structural modelling

The RosettaDock protocol from the Rosetta library [22] was used to model a dimer of the extracellular regions, starting with a monomer using the sequence of BG1 from the B12 haplotype modelled on the extracellular region of MOG (1PY9, [23]) as a template. An initial coarse round of docking was performed using randomized initial orientations of the docking partners with a harmonic distance constraint of 5.63 Å between the α-carbon atoms of Cys28. The lowest scoring structures, representing those complexes with the lowest calculated energy, from 100 000 models were assessed for viability. These had a high degree of similarity, so the lowest energy structure was used as an initial model for local refinement. During this step, the disulfide bond was enforced and built by RosettaDock using the relevant options, along with sidechain repacking. Plotting the score against root mean square deviation from the resulting 10 000 models revealed a population with a lower energy and high degree of similarity, indicative of the generation of a plausible model. The 15 models from this population were overlaid and the lowest energy model, presented as the main chain with the first carbon of the side chain, was used for the final pictures.

## 3. Results

### 3.1. BG0 is virtually monomorphic

**Figure 2.**
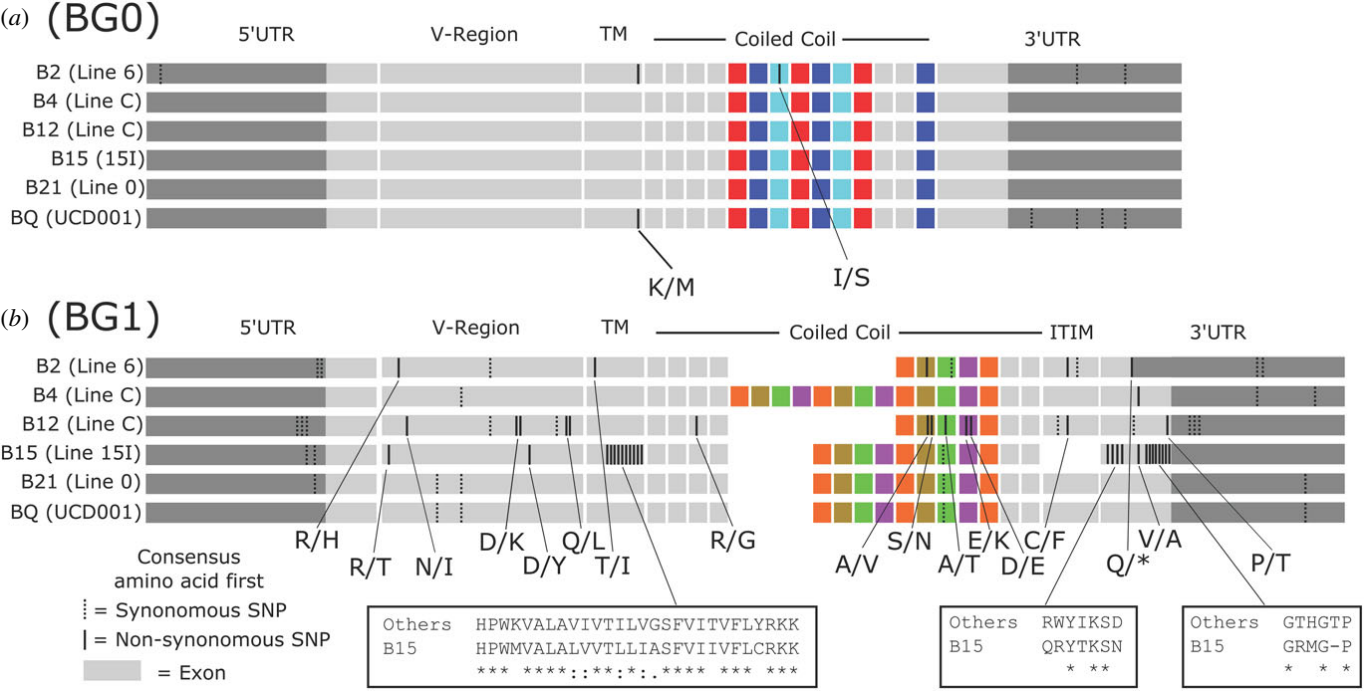
BG0 is nearly monomorphic, while BG1 is variable. Representation of alignments with consensus cDNA sequences from experimental chicken lines and predicted cDNA sequence from red jungle fowl genome. Non-coding sequence is indicated in dark grey, coding sequence in light grey or colours (same colour indicates same sequence, illustrating the repeating cytoplasmic exons; no cytoplasmic exons shared between BG0 and BG1), exon boundaries and missing exons are indicated by white lines and white regions. Synonymous differences from the consensus are indicated by dotted vertical lines; non-synonymous differences are indicated by solid vertical lines; sequence variation is shown below, with the consensus amino acid shown first and the variant amino acid shown second (detailed sequences in figures S2, S4 and [17]). (*a*) BG0 has only five non-coding and two non-synonymous nucleotide differences. (*b*) BG1 has SNPs throughout the sequence, concentrated in the V domain and 3′UTR with the most divergent B15 haplotype having clusters of differences, as well as CNV of cytoplasmic exon repeats.

We produced full-length BG0 cDNA sequences from spleen samples of various inbred experimental chicken lines derived from white leghorn egg-laying chickens, and compared them to the BG0 gene from the whole genome shotgun (WGS) sequence for line UCD001 derived from a red jungle fowl (figure 2*a*; electronic supplementary material, figure S1). Of the seven variable positions found, all are isolated single nucleotide polymorphisms (SNPs): one in the 5′UTR and four in the 3′UTR, a non-synonymous SNP at the intracellular end of the transmembrane region (Met in UCD001 and line 6_1_ versus Lys in the other lines) and another non-synonymous SNP in the cytoplasmic tail (Ser in line 6_1_ versus Ile in the other lines).

BG0 has a wide tissue distribution in C-B12 chickens [17], and so we examined full-length BG0 cDNA sequences derived from spleen, thymus, bone marrow, duodenum and kidney. A major transcript was present in all tissues and from all chicken lines, but some variant transcripts were found (electronic supplementary material, figures S2 and S3). Unlike other BG genes examined thus far, the 5′UTR is separated into two exons, the second of which includes the signal sequence. Four alternative splicing variants, totalling about 23% of sequences in all the chicken lines, were found flanking the major intron, ending before or at the start codon (electronic supplementary material, figures S1 and S2A). One alternative splicing variant located in the exon encoding the V domain was found in three lines, totalling about 10% of sequences, with the second intron finishing at a cryptic splice site near the end of the exon (electronic supplementary material, figure S2*b*). Finally, some variants were found with alternative exon splicing or intron read-through in the cytoplasmic tail (electronic supplementary material, figure S3A). Some read-throughs are located after the first or the last cytoplasmic exon, while all the others are located between the second and third exons of the three-exon repeat (coloured red–light blue–dark blue in figure 2*a*). Two skipped exons and one additional exon are also in the three-exon repeat region. Single examples of seven variants were found in one or another line, two examples of one variant were found in two lines, and six examples of another variant were found in three lines, totalling about 26% of sequences. Altogether, there were significant numbers of variant sequences, but no tissue-specific splicing or nucleotide variation was found, consonant with a housekeeping function for BG0.

### 3.2. BG1 is polymorphic, evolving mostly by point mutation except for copy number variation in the cytoplasmic tail

Full-length BG1 cDNA sequences were derived from kidney samples of the experimental chicken lines (as well as thymus, bone marrow and duodenum from line C-B12), and compared to the BG1 gene from line UCD001 (figure 2*b*; electronic supplementary material, S4). BG1 has variation throughout the sequence.

The most striking differences are in the length of the cytoplasmic tail, ranging from 13 exons in the B2 allele of line 6_1_ and the B12 allele of line C-B12 upwards to 21 exons in the B4 allele of line C-B4. For each haplotype, there was one predominant sequence with either no other sequences present or one example of a sequence with an intron read-through (electronic supplementary material, figure S3B). In addition, the first cytoplasmic exon was present in two forms in all but the B15 haplotype, either 21 or 24 nucleotides long (electronic supplementary material, figure S3C and S4). The sequences of the cytoplasmic exons are easily distinguishable, making it clear that virtually all the variation in exon number is due to expansion or contraction of a four-exon repeat, probably generated by unequal crossing-over in one exon (coloured orange in figure 2*b*). Of particular interest is the penultimate exon that contains an immunoreceptor tyrosine-based inhibitory motif (ITIM), which has been suggested to be functionally important [24] but which is missing in all sequences of the B15 allele. There is also one deleted codon in the final exon of the B15 allele.

All the other variation appears as SNPs due to point mutation, as shown by a *φ* test (*p* = 1.0). In the six alleles examined (excluding extra exons and intron read-throughs), there are six variable positions in the 5′UTR and seven in the 3′UTR. In the coding region, there are 39 non-synonymous nucleotide positions (leading to 29 amino acid changes) and nine synonymous positions, most of which are in the B15 sequence (electronic supplementary material, figure S4). There is no evidence for selection of variation overall (d*N*/d*S* = −0.761, *p* = 0.448) or in any portion of the sequence (V domain, −1.496, 0.137; transmembrane region, 0.845, 0.400; cytoplasmic tail, −1.226, 0.224). All the variable positions are spread out, except for the most divergent sequence from the B15 haplotype, which has one cluster of variation (10 nucleotides leading to eight amino acid changes over 22 positions) at the end of the transmembrane region and two clusters (four nucleotides leading to four amino acid changes over seven positions, and eight nucleotides leading to three amino acid changes over five positions) in the coding region of the final exon (figure 2; electronic supplementary material, S4). In addition, a SNP in the B2 sequence leads to a premature stop codon, removing 20 amino acids from the C-terminus of the protein.

**Figure 3.**
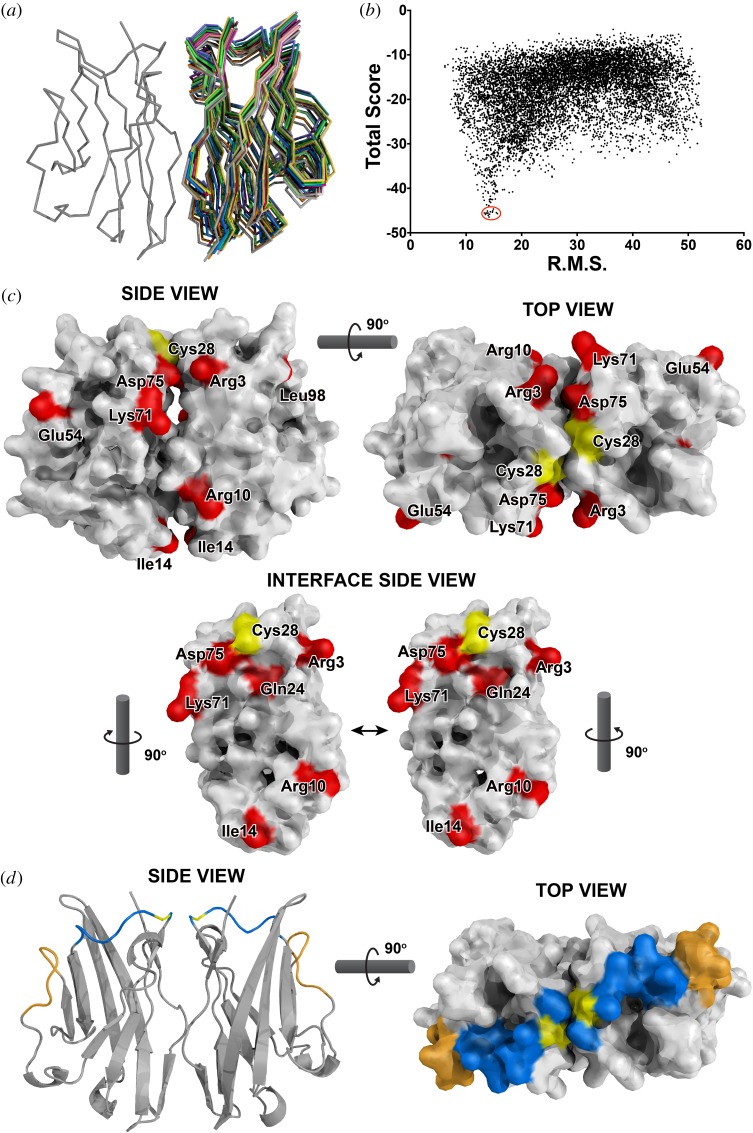
Structural models for BG V domain dimer suggest that extracellular sequence variation is found principally on the sides of the two V domains of BG1, and in a stripe across the top of the two domains comparing BG8, BG9 and BG12 to BG13. (*a*) Stick models of dimer represented by main chain atoms, with lowest energy configuration on left and best 15 models on right. (*b*) 10 000 models compared for root mean square deviation and for energy score, with best 15 models circled in red. (*c*) Space filling models of main chain and first carbon atom of the side chain as side view and top view of BG1 dimer, and side view of interface between the two domains with interchain disulfide residues in yellow and variant positions in red. Note the potential charge–charge interaction between Arg3 in one domain and Asp75 in the other domain. (*d*) Ribbon model of side view and space filling model of main chain and first carbon atom of the side chain as top view of BG dimers, with interchain disulfide residues in yellow and positions different between BG8, BG9 and BG12 compared with BG13 in blue (residues 26–34 of the mature protein) and orange (residues 51–57).

None of the extracellular sequences differed by more than three amino acids from the consensus. Modelling a dimer of the extracellular domains (constrained by the interchain disulfide between cysteines located in the loop corresponding to CDR1 of antibodies) showed that the two V-like domains are likely to interact through their four-strand faces (figure 3*a*–*c*), as suggested previously [25]. All the variable positions are located in or near the interface of the two domains except one near the membrane (figure 3*c*), suggesting that recognition of the extracellular domains is likely to be by conserved ligand(s) or receptor(s).

### 3.3. Some BG genes in the BG region swap cassettes in loops corresponding to CDR1 and CDR2

In phylogenetic analysis, the alleles of the singleton genes BG0 and BG1 form separate clusters, which are also separate from the clusters formed by the BG genes of the BG region (figure 1*b*). The BG region genes are arrayed in tandem and in the same transcriptional orientation, and show all the signs of CNV by unequal crossing-over and/or deletion, including the presence of hybrid genes. Phylogenetic trees of the 5′ and 3′ ends show clear clusters with long branches and good bootstrap support, while phylogenetic trees of the extracellular region generally are not well resolved, with short branches and poor bootstrap support [17]. For these reasons, it is not yet clear which genes in different BG haplotypes may be thought of as alleles.

**Figure 4.**
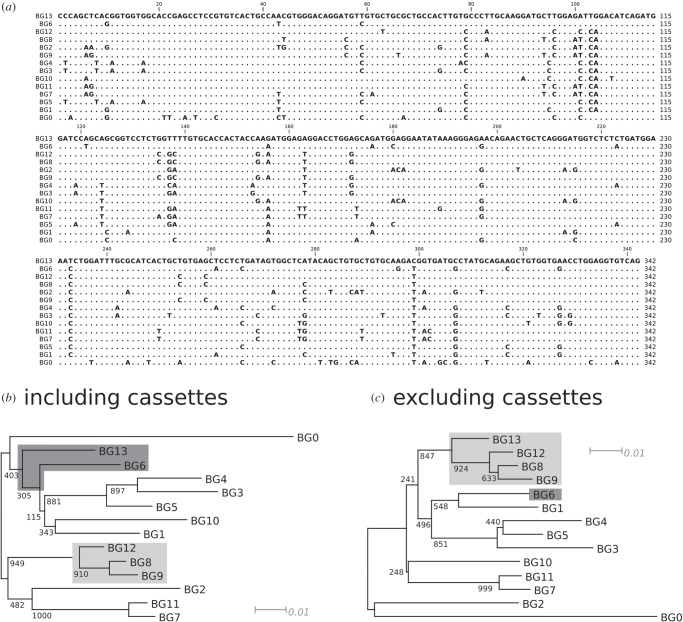
Phylogenetic comparisons of BG genes with and without the two cassettes encoding the loops corresponding to CDR1 and CDR2 illustrate segmental exchange. (*a*) Alignment of nucleotide sequences of the V domain exon of the 14 BG genes of the B12 haplotype used as the basis for NJ trees (with genetic distance indicated, and bootstrap values at the branch points). (*b*) Tree with the complete V domain sequence, showing that BG13 clusters with BG6 (highlighted in dark grey). (*c*) Tree with the V domain sequence but with the two cassettes deleted, showing that BG13 clusters with BG8, BG9 and BG12 (highlighted in light grey). Based on these trees, a similar relationship might describe BG1, BG6 and BG10.

**Figure 5.**
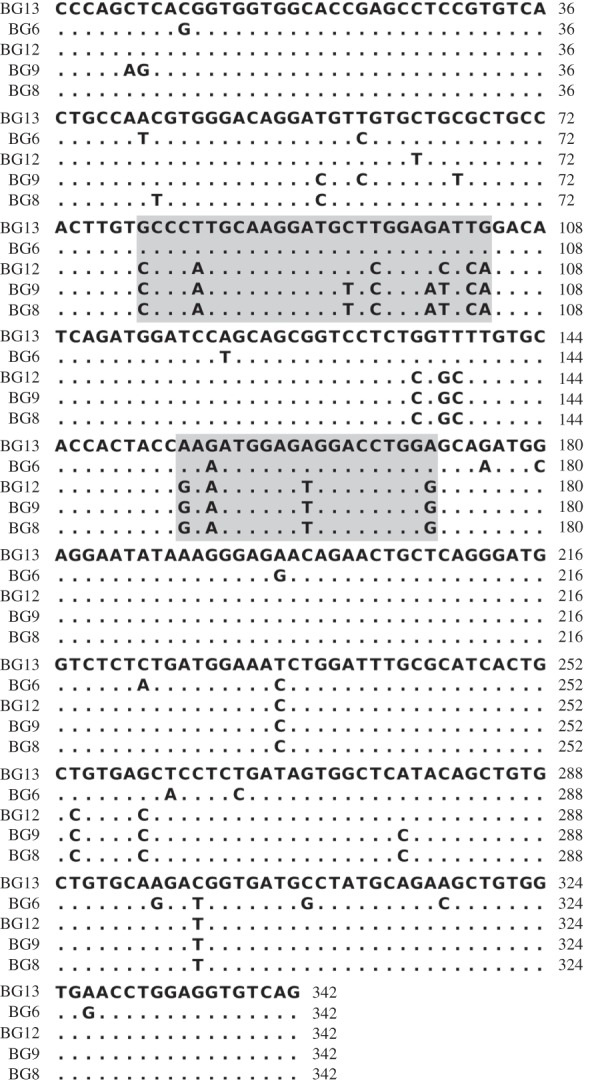
Segmental exchange found between BG genes in the BG region. Alignment of nucleotide sequences encoding the V domains of BG6, BG8, BG9, BG12 and BG13, with numbering relative to the start of the V domain sequence [17]. The two cassettes are highlighted with grey boxes.

**Figure 6.**
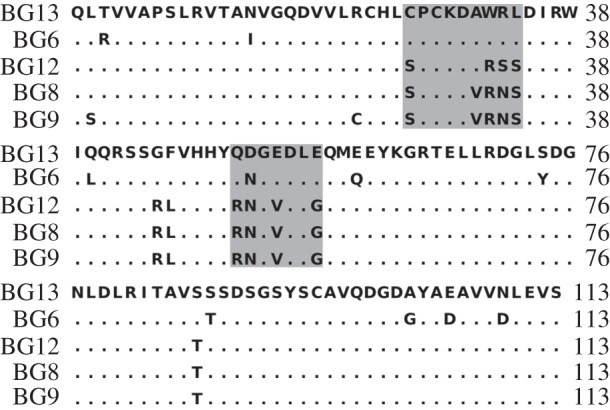
Segmental exchange found between BG genes in the BG region. Alignment of amino acid sequences encoding the V domains of BG6, BG8, BG9, BG12 and BG13, with numbering relative to the start of the V domain sequence [17]. The two cassettes are highlighted with grey boxes; note that the second cassette could be considered to start at amino acid position 45 and end at position 57, or even that both cassettes are part of a single segment starting before position 26 and ending just after position 57.

Within the B12 haplotype, BG8, BG9, BG12 and BG13 genes are overall closely related and expressed in the same kinds of cells and tissues [17]. In the extracellular region, BG8, BG9 and BG12 differ only in scattered nucleotides and amino acids, while BG13 is quite distant, located very far away in phylogenetic analysis (figure 4*a*,*b*). Most of the differences between the extracellular region of BG13 and the extracellular regions of BG8, BG9 and BG12 are found in two clusters: one with eight nucleotide positions leading to five differences within nine amino acids, and the other with four nucleotide positions leading to four differences within seven amino acids (figures 5 and 6). Such concentration of differences is statistically unlikely to have arisen by point mutation (*φ* test, *p* = 0.0017), and removal of these two stretches of sequence renders BG13 very similar to the other three extracellular regions in phylogenetic analysis (figure 4*c*). Comparison with the sequences of other BG genes (figures 4–6) shows that these two stretches of sequences in BG13 match those in the BG6 gene (except for one nucleotide), suggesting that these two stretches of sequences originated from micro-recombination (either by double reciprocal recombination or by gene conversion) between these genes.

In a model of the dimer of BG extracellular domains, these two stretches of sequence are located in the loops corresponding to the CDR1 and CDR2 of antibody V regions (figure 3*d*). Together, they form a stripe across the top of the dimer, suggesting that they are involved in binding ligands whose sequences vary, much like the way antibodies bind antigens.

## 4. Discussion

Attempting to understand the apparent differences in the tempo of evolution between mammalian butyrophilin and chicken BG genes, we examined full-length sequences from alleles and loci of the three BG lineages. We find different modes of evolution for these three lineages, which may be understood by their genomic locations and may also provide clues to their potential functions.

BG0 is a singleton gene found on chromosome 2 (around 97.95 Mb in current ENSEMBL assembly, Galgal4), surrounded by unrelated genes with typical cellular housekeeping functions (such as N-ethylmaleimide-sensitive factor attachment protein, gamma (NAPG), piezo-type mechanosensitive ion channel component 2 (PIEZO2), adenomatosis polyposis coli downregulated 1 (APCDD1), VAMP-associated protein A (VAPA), RAB31 and protein phosphatase 4 regulatory subunit 1 (PPP4R1)). No gene similar to BG0 was found in the syntenic region with these genes on human chromosome 18 (around 9–11 Mb in current ENSEMBL assembly). We found just a few synonymous nucleotide changes and only two amino acid changes in the predominant BG0 sequences from six white leghorn chicken lines. This strong conservation of sequence suggests purifying selection for some important, presumably housekeeping, function. Alternatively, BG0 might act as a conserved partner in a heterodimer with other proteins that are polymorphic, such as the products of other BG genes. At the moment, the evidence for homodimers versus heterodimers is not conclusive [15,26].

Interestingly, about 20% of the BG0 transcripts had alternative splicing in the 5‘UTR (just before the start codon) and about 10% had alternative splicing removing most of the extracellular V region. These occurrences of the alternative splicing are not correlated with each other, haplotype or tissue examined, so the phenomena remain mysteries but might point to differences in regulation or in function.

BG1 is also a singleton gene but located in the BF-BL region (the chicken MHC) filled with highly polymorphic genes. Each of the six haplotypes has a different allele of BG1, but the few SNPs are scattered along the sequences (except for the B15 haplotype, which had several clusters of variation). There was no evidence for selection of variation anywhere along the sequence, including the extracellular V-like regions, which have at most three amino acid differences from the consensus. Modelling suggests that the few differences straggle down the sides of the BG1 dimer, located around the interface of the two domains, and away from the membrane-distal top of the molecule. Taken together, these data suggest that interactions with receptors (for instance, semi-invariant γδ TCR as in some Btns) or ligands outside the cell are likely to be conserved rather than variable, with the small variations in the V-like domain due to drift or to genetic hitchhiking on the highly polymorphic MHC genes nearby.

However, two kinds of major differences between BG1 alleles were found in the cytoplasmic tail, which is composed of seven amino acid repeats presumably forming an α-helical coiled coil between the two subunits of the dimer. The transcripts varied between one and three copies of a four-exon repeat. This expansion and contraction has also been reported at the DNA level [19], suggesting that unequal crossing-over is responsible, rather than differential expression of alternatively spliced transcripts. Another variation was the presence of the penultimate exon, which was not found in any transcript from the B15 haplotype. Genomic sequence from another B15 chicken also shows the deletion of this exon [19]. This penultimate exon is composed of two 21 nucleotide repeat exons flanking a read-through intron [17], with the read-through encoding an apparently functional ITIM, which is suggested to contribute to resistance against viral diseases [24]. The potential importance of cytoplasmic tails in BG function is also suggested by the discovery of the ‘zipper protein’ [27]. However, it is not yet clear whether the differences in the four-exon repeats or the penultimate exon in the cytoplasmic tail of the BG1 gene have been selected, or are just genomic accidents.

In contrast with BG0 and BG1, the other BG genes are present in the BG region as a tandem array in the same transcriptional orientation. Such a genomic arrangement would allow unequal crossing-over and deletion resulting in CNV, and indeed these BG genes show all the expected features including variable numbers of hybrid genes [17]. Such variation makes it difficult to establish which genes are orthologous, which in turn makes it difficult to compare alleles in order to establish which portions of the sequence are functionally important.

However, the proximity of many related genes would favour gene conversion and/or double reciprocal recombination (also referred to as ‘segmental exchange’ or ‘micro-recombination’) leading to sharing of short sequence stretches (as discussed in [28,29]). Many MHC class I and class II genes have been diversified in this manner, and were then selected for recognition of peptides as part of the process of responding to pathogens and tumours ([30–32], but see [33]). Within the BG region of the B12 haplotype is an example of such segmental exchange, in which two short sequences from the donor gene BG6 replaced homologous regions in a family of closely related genes including BG8, BG9 and BG12 to generate the new gene BG13. Interestingly, the exchanges occurred in the sequence encoding the loops corresponding to CDR1 and CDR2 in antibodies and TCR. Given that the extracellular V-like domains of the BG dimer must be oriented so that the four-strand face is the contact, molecular modelling places the variable sequence in a stripe that crosses the centre of the most membrane-distal part of the molecule. This variation in a region well placed to interact with extracellular ligands or receptors argues that the interacting molecule will also be variable. Such a situation would be selected if these BG molecules were involved in recognition of pathogens, or if they were ligands for variable receptors (such as γδ TCRs, as for some Btn molecules, reviewed in [6–9]). However, another possibility is that the level of segmental exchange results in a high mutation rate, diversifying the BG genes in the BG region and contributing to the serological polymorphism without any selection. These possibilities may be distinguished once functional alleles in different BG haplotypes are determined.

The three lineages of BG genes vary in their mode of evolution based on their genomic environment. The singleton BG0 seems nearly invariant (although with potentially interesting alternative splicing variants) with a presumed housekeeping function. The singleton BG1 in the BF-BL region of the MHC has some variation by point mutation that does not appear to be selected for diversity, so the extracellular domains do not appear to be involved in binding variable ligands. However, there is significant variation in the presence of exons in the cytoplasmic tail, with potentially important significance [25]. Finally, in the tandem array of BG genes in the BG region, at least one gene has undergone segmental exchange in loops likely to be involved in recognition of ligands. As the recipient gene is otherwise closely related to other genes with similar cell and tissue expression patterns, it seems likely that all these genes perform similar functions and that the sequence segments exchanged are selected for variation. If so, such BG genes have the characteristics expected for function within the immune response.

There seem to be no direct correspondences between BG genes and their mammalian homologues such as Btn, Btn-like and SKINT genes, as assessed both by phylogenetic analysis [1] and by inspection of the available genomes (www.ensembl.ac.uk). BG1 (and a chicken Btn gene predicted by bioinformatics) are located at the edge of the class II region of the MHC, as are mammalian Btn-like genes, one in humans (along with three on chromosome 5) and four in mice (with two pseudogenes and on chromosome 11 another intact gene). The BG genes in the BG region might correspond to MOG in the extended class I region in both humans and mice (along with two Btn genes on human chromosome 13 and on mouse chromosome 13). However, there does not seem to be a homolog of BG0 in the syntenic regions on human chromosome 18 or mouse chromosome 17, the SKINT and ERMAP genes on human chromosome 1 and mouse chromosome 4 are not obviously represented in chickens, and the chicken Btn gene Tva1 located on chicken chromosome 28 is not obviously present in the syntenic regions on human chromosome 19 and mouse chromosome 17.

However, it seems that the functional constraints and evolutionary processes we have found in BG genes may be mirrored in some of the human homologues. The involvement of Btn1A1 in the structure of lipid droplets in milk would not obviously require enormous diversity, which is much like BG0 and perhaps like BG1. By contrast, the functions currently ascribed to Btn and Btn-like genes in T cell function might evolve variation in response to pathogen immune evasion, and several of these genes bear SNPs with impact at least upon autoimmune diseases. However, there are no parallels with the rapid evolution found in the BG genes of the BG region, and no examples of segmental exchange are reported as we find for some BG genes. Thus, some BG genes may have functions (such as direct pathogen recognition) that require very rapid evolution.

## Supplementary Material

Chattaway et al Supplementary figures with legends

## Data accessibility

DNA sequences: final DNA sequences as alignments uploaded as electronic supplementary material, and GenBank accessions nos. KC955130, KC955131, KC963427 and KX686497-KX686505.
