# Supplementary material for: Different modes of variation for each BG lineage suggest different functions
Source: Open Biol. 2016 Sep 14;6(9):160188. doi: 10.1098/rsob.160188 (PMC5043582; doi:10.1098/rsob.160188)
Supplement: Chattaway et al Supplementary figures with legends [file rsob160188supp1.pdf]

Chattaway J, Ramirez-Valdez RA, Chappell PE, Caesar JJE, Lea SM and Kaufman J.
Different modes of variation for each BG lineage suggest different functions. 2016. Open Biology
Electronic Supplementary Material

```
BG0-B2   TGCACCACCAGAGAGGACAGCCGGAAGGTCTGCACTGCTCTCCTTCAGCTTCTTAGAGCTCCTTTTTGCTTCTTCCCCTCTCCTCTTCTCCAGCACAGAT 100
ORF/CDS                                                                                                     M
BG0-B4   TGCACCACCAGAGAGGACAGCCAGAAGGTCTGCACTGCTCTCCTTCAGCTTCTTAGAGCTCCTTTTTGCTTCTTCCCCTCTCCTCTTCTCCAGCACAGAT 100
ORF/CDS                                                                                                     M
BG0-B12  TGCACCACCAGAGAGGACAGCCAGAAGGTCTGCACTGCTCTCCTTCAGCTTCTTAGAGCTCCTTTTTGCTTCTTCCCCTCTCCTCTTCTCCAGCACAGAT 100
ORF/CDS                                                                                                     M
BG0-B15  TGCACCACCAGAGAGGACAGCCAGAAGGTCTGCACTGCTCTCCTTCAGCTTCTTAGAGCTCCTTTTTGCTTCTTCCCCTCTCCTCTTCTCCAGCACAGAT 100
ORF/CDS                                                                                                     M
BG0-B21  TGCACCACCAGAGAGGACAGCCAGAAGGTCTGCACTGCTCTCCTTCAGCTTCTTAGAGCTCCTTTTTGCTTCTTCCCCTCTCCTCTTCTCCAGCACAGAT 100
ORF/CDS                                                                                                     M
BG0-BQ   TGCACCACCAGAGAGGACAGCCAGAAGGTCTGCACTGCTCTCCTTCAGCTTCTTAGAGCTCCTTTTTGCTTCTTCCCCTCTCCTCTTCTCCAGCACAGAT 100
ORF/CDS                                                                                                     M

BG0-B2   GTGGTTCGTATCAGGCTGCAAACAGTCCAGTTTCGCCCTCCCCTGGAGGACCCTCCTGGCTTACCTCGTGGCTCTCAACCTCCTCTGTCCAGGATCAGCC 200
ORF/CDS    W  F  V  S  G  C  K  Q  S  S  F  A  L  P  W  R  T  L  L  A  Y  L  V  A  L  N  L  L  C  P  G  S  A
BG0-B4   GTGGTTCGTATCAGGCTGCAAACAGTCCAGTTTCGCCCTCCCCTGGAGGACCCTCCTGGCTTACCTCGTGGCTCTCAACCTCCTCTGTCCAGGATCAGCC 200
ORF/CDS    W  F  V  S  G  C  K  Q  S  S  F  A  L  P  W  R  T  L  L  A  Y  L  V  A  L  N  L  L  C  P  G  S  A
BG0-B12  GTGGTTCGTATCAGGCTGCAAACAGTCCAGTTTCGCCCTCCCCTGGAGGACCCTCCTGGCTTACCTCGTGGCTCTCAACCTCCTCTGTCCAGGATCAGCC 200
ORF/CDS    W  F  V  S  G  C  K  Q  S  S  F  A  L  P  W  R  T  L  L  A  Y  L  V  A  L  N  L  L  C  P  G  S  A
BG0-B15  GTGGTTCGTATCAGGCTGCAAACAGTCCAGTTTCGCCCTCCCCTGGAGGACCCTCCTGGCTTACCTCGTGGCTCTCAACCTCCTCTGTCCAGGATCAGCC 200
ORF/CDS    W  F  V  S  G  C  K  Q  S  S  F  A  L  P  W  R  T  L  L  A  Y  L  V  A  L  N  L  L  C  P  G  S  A
BG0-B21  GTGGTTCGTATCAGGCTGCAAACAGTCCAGTTTCGCCCTCCCCTGGAGGACCCTCCTGGCTTACCTCGTGGCTCTCAACCTCCTCTGTCCAGGATCAGCC 200
ORF/CDS    W  F  V  S  G  C  K  Q  S  S  F  A  L  P  W  R  T  L  L  A  Y  L  V  A  L  N  L  L  C  P  G  S  A
BG0-BQ   GTGGTTCGTATCAGGCTGCAAACAGTCCAGTTTCGCCCTCCCCTGGAGGACCCTCCTGGCTTACCTCGTGGCTCTCAACCTCCTCTGTCCAGGATCAGCC 200
ORF/CDS    W  F  V  S  G  C  K  Q  S  S  F  A  L  P  W  R  T  L  L  A  Y  L  V  A  L  N  L  L  C  P  G  S  A

BG0-B2   AAGCTCAGGGTGGTGGCATTGAACTTCCCTGTCACTGCCACTGTGGGACAGGATGTCGTGCTGCACTGCCACTTGTCCCCTTGCAAGGATGCTCGGAGCT 300
ORF/CDS   K  L  R  V  V  A  L  N  F  P  V  T  A  T  V  G  Q  D  V  V  L  H  C  H  L  S  P  C  K  D  A  R  S
BG0-B4   AAGCTCAGGGTGGTGGCATTGAACTTCCCTGTCACTGCCACTGTGGGACAGGATGTCGTGCTGCACTGCCACTTGTCCCCTTGCAAGGATGCTCGGAGCT 300
ORF/CDS   K  L  R  V  V  A  L  N  F  P  V  T  A  T  V  G  Q  D  V  V  L  H  C  H  L  S  P  C  K  D  A  R  S
BG0-B12  AAGCTCAGGGTGGTGGCATTGAACTTCCCTGTCACTGCCACTGTGGGACAGGATGTCGTGCTGCACTGCCACTTGTCCCCTTGCAAGGATGCTCGGAGCT 300
ORF/CDS   K  L  R  V  V  A  L  N  F  P  V  T  A  T  V  G  Q  D  V  V  L  H  C  H  L  S  P  C  K  D  A  R  S
BG0-B15  AAGCTCAGGGTGGTGGCATTGAACTTCCCTGTCACTGCCACTGTGGGACAGGATGTCGTGCTGCACTGCCACTTGTCCCCTTGCAAGGATGCTCGGAGCT 300
ORF/CDS   K  L  R  V  V  A  L  N  F  P  V  T  A  T  V  G  Q  D  V  V  L  H  C  H  L  S  P  C  K  D  A  R  S
BG0-B21  AAGCTCAGGGTGGTGGCATTGAACTTCCCTGTCACTGCCACTGTGGGACAGGATGTCGTGCTGCACTGCCACTTGTCCCCTTGCAAGGATGCTCGGAGCT 300
ORF/CDS   K  L  R  V  V  A  L  N  F  P  V  T  A  T  V  G  Q  D  V  V  L  H  C  H  L  S  P  C  K  D  A  R  S
BG0-BQ   AAGCTCAGGGTGGTGGCATTGAACTTCCCTGTCACTGCCACTGTGGGACAGGATGTCGTGCTGCACTGCCACTTGTCCCCTTGCAAGGATGCTCGGAGCT 300
ORF/CDS   K  L  R  V  V  A  L  N  F  P  V  T  A  T  V  G  Q  D  V  V  L  H  C  H  L  S  P  C  K  D  A  R  S

BG0-B2   TGGACATCAGATGGATCCAGCACCGGTCCTCTGGTCTTGTGCACCACTACCAAAATGGAGAGGACCTGGAACAGATGGAGGAATATAAAGGGAGGACAGA 400
ORF/CDS  L  D  I  R  W  I  Q  H  R  S  S  G  L  V  H  H  Y  Q  N  G  E  D  L  E  Q  M  E  E  Y  K  G  R  T  E
BG0-B4   TGGACATCAGATGGATCCAGCACCGGTCCTCTGGTCTTGTGCACCACTACCAAAATGGAGAGGACCTGGAACAGATGGAGGAATATAAAGGGAGGACAGA 400
ORF/CDS  L  D  I  R  W  I  Q  H  R  S  S  G  L  V  H  H  Y  Q  N  G  E  D  L  E  Q  M  E  E  Y  K  G  R  T  E
BG0-B12  TGGACATCAGATGGATCCAGCACCGGTCCTCTGGTCTTGTGCACCACTACCAAAATGGAGAGGACCTGGAACAGATGGAGGAATATAAAGGGAGGACAGA 400
ORF/CDS  L  D  I  R  W  I  Q  H  R  S  S  G  L  V  H  H  Y  Q  N  G  E  D  L  E  Q  M  E  E  Y  K  G  R  T  E
BG0-B15  TGGACATCAGATGGATCCAGCACCGGTCCTCTGGTCTTGTGCACCACTACCAAAATGGAGAGGACCTGGAACAGATGGAGGAATATAAAGGGAGGACAGA 400
ORF/CDS  L  D  I  R  W  I  Q  H  R  S  S  G  L  V  H  H  Y  Q  N  G  E  D  L  E  Q  M  E  E  Y  K  G  R  T  E
BG0-B21  TGGACATCAGATGGATCCAGCACCGGTCCTCTGGTCTTGTGCACCACTACCAAAATGGAGAGGACCTGGAACAGATGGAGGAATATAAAGGGAGGACAGA 400
ORF/CDS  L  D  I  R  W  I  Q  H  R  S  S  G  L  V  H  H  Y  Q  N  G  E  D  L  E  Q  M  E  E  Y  K  G  R  T  E
BG0-BQ   TGGACATCAGATGGATCCAGCACCGGTCCTCTGGTCTTGTGCACCACTACCAAAATGGAGAGGACCTGGAACAGATGGAGGAATATAAAGGGAGGACAGA 400
ORF/CDS  L  D  I  R  W  I  Q  H  R  S  S  G  L  V  H  H  Y  Q  N  G  E  D  L  E  Q  M  E  E  Y  K  G  R  T  E

BG0-B2   ACTGCTCAGGGACGGTCTCTCTGATGGAAATCTGTATTTGCACATTACTGCAGTGAGCTCCTCCGATAGCGGCTCATATATGTGCACTGTGCAAGATGAT 500
ORF/CDS    L  L  R  D  G  L  S  D  G  N  L  Y  L  H  I  T  A  V  S  S  S  D  S  G  S  Y  M  C  T  V  Q  D  D
BG0-B4   ACTGCTCAGGGACGGTCTCTCTGATGGAAATCTGTATTTGCACATTACTGCAGTGAGCTCCTCCGATAGCGGCTCATATATGTGCACTGTGCAAGATGAT 500
ORF/CDS    L  L  R  D  G  L  S  D  G  N  L  Y  L  H  I  T  A  V  S  S  S  D  S  G  S  Y  M  C  T  V  Q  D  D
BG0-B12  ACTGCTCAGGGACGGTCTCTCTGATGGAAATCTGTATTTGCACATTACTGCAGTGAGCTCCTCCGATAGCGGCTCATATATGTGCACTGTGCAAGATGAT 500
ORF/CDS    L  L  R  D  G  L  S  D  G  N  L  Y  L  H  I  T  A  V  S  S  S  D  S  G  S  Y  M  C  T  V  Q  D  D
BG0-B15  ACTGCTCAGGGACGGTCTCTCTGATGGAAATCTGTATTTGCACATTACTGCAGTGAGCTCCTCCGATAGCGGCTCATATATGTGCACTGTGCAAGATGAT 500
ORF/CDS    L  L  R  D  G  L  S  D  G  N  L  Y  L  H  I  T  A  V  S  S  S  D  S  G  S  Y  M  C  T  V  Q  D  D
BG0-B21  ACTGCTCAGGGACGGTCTCTCTGATGGAAATCTGTATTTGCACATTACTGCAGTGAGCTCCTCCGATAGCGGCTCATATATGTGCACTGTGCAAGATGAT 500
ORF/CDS    L  L  R  D  G  L  S  D  G  N  L  Y  L  H  I  T  A  V  S  S  S  D  S  G  S  Y  M  C  T  V  Q  D  D
BG0-BQ   ACTGCTCAGGGACGGTCTCTCTGATGGAAATCTGTATTTGCACATTACTGCAGTGAGCTCCTCCGATAGCGGCTCATATATGTGCACTGTGCAAGATGAT 500
ORF/CDS    L  L  R  D  G  L  S  D  G  N  L  Y  L  H  I  T  A  V  S  S  S  D  S  G  S  Y  M  C  T  V  Q  D  D
```

Fig. S1, continued next page.

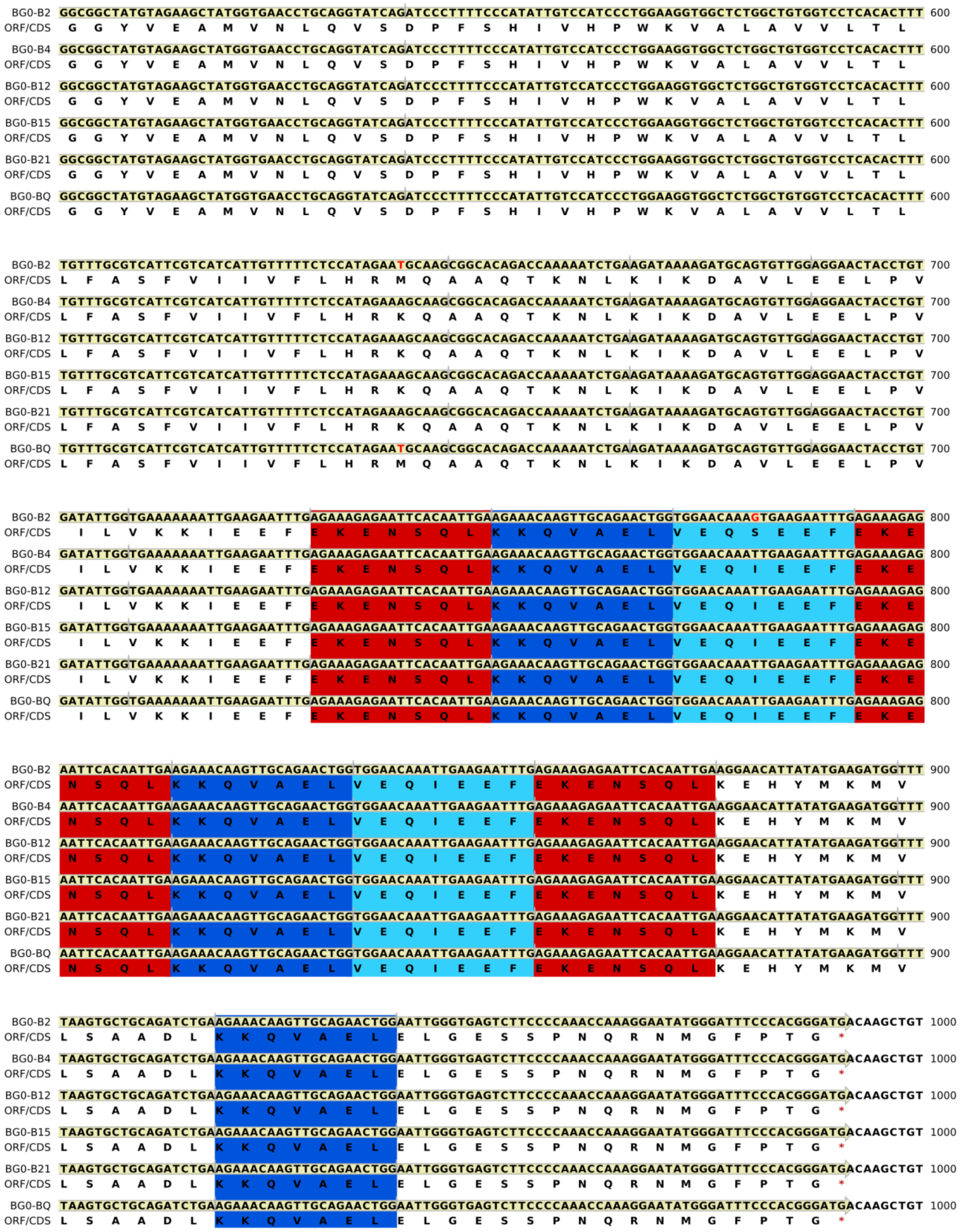

Fig. S1, continued next page.

```
 BG0-B2 CCCTCCTCAGCTTCCGTTGCTTTTCTCTTTCTTTTCCTGGAAAACAATTTGAAGAATCAGATTGAGAAATGAATTGTGCCTCGCAGTAACCACAGGTTTC 1100
ORF/CDS
 BG0-B4 CCCTCCTCAGCTTCCGTTGCTTTTCTCTTTCTTTTCCTGGAAAACAATTTGAAGAATCAGATTGAGAAATGAATTGTGCCTCGCAGTAACCACAGGTTTC 1100
ORF/CDS
BG0-B12 CCCTCCTCAGCTTCCGTTGCTTTTCTCTTTCTTTTCCTGGAAAACAATTTGAAGAATCAGATTGAGAAATGAATTGTGCCTCGCAGTAACCACAGGTTTC 1100
ORF/CDS
BG0-B15 CCCTCCTCAGCTTCCGTTGCTTTTCTCTTTCTTTTCCTGGAAAACAATTTGAAGAATCAGATTGAGAAATGAATTGTGCCTCGCAGTAACCACAGGTTTC 1100
ORF/CDS
BG0-B21 CCCTCCTCAGCTTCCGTTGCTTTTCTCTTTCTTTTCCTGGAAAACAATTTGAAGAATCAGATTGAGAAATGAATTGTGCCTCGCAGTAACCACAGGTTTC 1100
ORF/CDS
 BG0-BQ CCCTCCTCAGCTTCCATTGCTTTTCTCTTTCTTTTCCTGGAAAACAATTTGAAGAATCAGATTGAGAAATGAATTGTGCCTCGCAGTAACCACAGGTTTC 1100
ORF/CDS

 BG0-B2 AAGCTTACTAGACTGCTGATTGCATAGGACATCAAAACCCTGATAACTTGAAGCAGGCAATGAAAACCACAAGGGGAACAAGACGAGCTAGTGTTTACAT 1200
ORF/CDS
 BG0-B4 AAGCTTACTAGACTACTGATTGCATAGGACATCAAAACCCTGATAACTTGAAGCAGGCAATGAAAACCACAAGGGGAACAAGACGAGCTAGTGTTTACAT 1200
ORF/CDS
BG0-B12 AAGCTTACTAGACTACTGATTGCATAGGACATCAAAACCCTGATAACTTGAAGCAGGCAATGAAAACCACAAGGGGAACAAGACGAGCTAGTGTTTACAT 1200
ORF/CDS
BG0-B15 AAGCTTACTAGACTACTGATTGCATAGGACATCAAAACCCTGATAACTTGAAGCAGGCAATGAAAACCACAAGGGGAACAAGACGAGCTAGTGTTTACAT 1200
ORF/CDS
BG0-B21 AAGCTTACTAGACTACTGATTGCATAGGACATCAAAACCCTGATAACTTGAAGCAGGCAATGAAAACCACAAGGGGAACAAGACGAGCTAGTGTTTACAT 1200
ORF/CDS
 BG0-BQ AAGCTTACTAGACTGCTGATTGCATAGGACATCAAAACCCTGATAACTTGAAGCAGGCAATGAAAACCAGAAGGGGAACAAGACGAGCTAGTGTTTACAT 1200
ORF/CDS

 BG0-B2 TGAGTGAGAACACCTGCATTTTTGTGACCAAAAATTGCATGAAGGGCTACCGAACTGAGGATGCATGAACTCCAACTCATATTCAGCTGGAATAAAGAAA 1300
ORF/CDS
 BG0-B4 TGAGTGAGAACACCTGCATTTTTGTGACCAAAAATTGCATGAAGGGCTACCGAACAGAGGATGCATGAACTCCAACTCATATTCAGCTGGAATAAAGAAA 1300
ORF/CDS
BG0-B12 TGAGTGAGAACACCTGCATTTTTGTGACCAAAAATTGCATGAAGGGCTACCGAACAGAGGATGCATGAACTCCAACTCATATTCAGCTGGAATAAAGAAA 1300
ORF/CDS
BG0-B15 TGAGTGAGAACACCTGCATTTTTGTGACCAAAAATTGCATGAAGGGCTACCGAACAGAGGATGCATGAACTCCAACTCATATTCAGCTGGAATAAAGAAA 1300
ORF/CDS
BG0-B21 TGAGTGAGAACACCTGCATTTTTGTGACCAAAAATTGCATGAAGGGCTACCGAACAGAGGATGCATGAACTCCAACTCATATTCAGCTGGAATAAAGAAA 1300
ORF/CDS
 BG0-BQ TGAGTGAGAACACCTGCATTTTTGTGACCAAAAATTGCATGAAGGGCTACCGAACTGAGGATGCATGAACTCCAACTCATATTCAGCTGGAATAAAGAAA 1300
ORF/CDS

 BG0-B2 CCACATAAGGAAGAAAATGGGTTGAAGATGGAGTTCCTGGAAGAGACAGAAATTTTGGGAAATAGTATGATCATCTATCAAGCTTCATAGAAATCTAATG 1400
ORF/CDS
 BG0-B4 CCACATAAGGAAGAAAATGGGTTGAAGATGGAGTTCCTGGAAGAGACAGAAATTTTGGGAAATAGTATGATCATCTATCAAGCTTCATAGAAATCTAATG 1400
ORF/CDS
BG0-B12 CCACATAAGGAAGAAAATGGGTTGAAGATGGAGTTCCTGGAAGAGACAGAAATTTTGGGAAATAGTATGATCATCTATCAAGCTTCATAGAAATCTAATG 1400
ORF/CDS
BG0-B15 CCACATAAGGAAGAAAATGGGTTGAAGATGGAGTTCCTGGAAGAGACAGAAATTTTGGGAAATAGTATGATCATCTATCAAGCTTCATAGAAATCTAATG 1400
ORF/CDS
BG0-B21 CCACATAAGGAAGAAAATGGGTTGAAGATGGAGTTCCTGGAAGAGACAGAAATTTTGGGAAATAGTATGATCATCTATCAAGCTTCATAGAAATCTAATG 1400
ORF/CDS
 BG0-BQ CCACATAAGGAAGAAAATGGGTTGAAGATGGAGTTCCTGGAAGAGACAGAAATTTTGGGAAATAGTATGATCATCTATCAAGCTTCATAGAAATCTAATG 1400
ORF/CDS

 BG0-B2 AATATGTAATGCTTTTGGAAACACAAGCATGCATATCGAAGCAAAGACAGAAAACTGCTTTGGGTGTTAATCCCATTG 1478
ORF/CDS
 BG0-B4 AATATGTAATGCTTTTGGAAACACAAGCATGCATATCGAAGCAAAGACAGAAAACTGCTTTGGGTGTTAATCCCATTG 1478
ORF/CDS
BG0-B12 AATATGTAATGCTTTTGGAAACACAAGCATGCATATCGAAGCAAAGACAGAAAACTGCTTTGGGTGTTAATCCCATTG 1478
ORF/CDS
BG0-B15 AATATGTAATGCTTTTGGAAACACAAGCATGCATATCGAAGCAAAGACAGAAAACTGCTTTGGGTGTTAATCCCATTG 1478
ORF/CDS
BG0-B21 AATATGTAATGCTTTTGGAAACACAAGCATGCATATCGAAGCAAAGACAGAAAACTGCTTTGGGTGTTAATCCCATTG 1478
ORF/CDS
 BG0-BQ AATATGTAATGCTTTTGGAAACACAAGCATGCATATCGAAGCAAAGACAGAAAACTGCTTTGGGTGTTAATCCCATTG 1478
ORF/CDS
```

Figure S1. Nucleotide and protein sequence alignment of the consensus BG0 cDNAs from each haplotype. Position numbers are relative to the start of the 5' primer binding site, which covers up to position 22. The 3' primer binding site starts at position 1454. Consensus is defined as containing the exons for the 5'UTR, V domain, transmembrane region, all of the cytoplasmic tail, and the 3'UTR, but conditionally expressed indels and read-through exons are excluded. Open reading frames are highlighted in yellow, and protein sequence is displayed below the open reading frame. Exons are denoted by faint grey ticks above the coding sequence. Short cytoplasmic exons are coloured with the same colours as in Fig. 1. Stop codons are shown as asterisks, gaps are shown as dashes, and polymorphic residues are highlighted in red. These sequences were deposited in Genbank (with the following accession numbers): BG0C (KX686497), BG015I (KX686498), BG0N (KX686499), BG061 (KX686500). (Chattaway J et al. 2016. Open Biology)

Chattaway J, Ramirez-Valdez RA, Chappell PE, Caesar JJE, Lea SM and Kaufman J.
Different modes of variation for each BG lineage suggest different functions. 2016. Open Biology
Electronic Supplementary Material

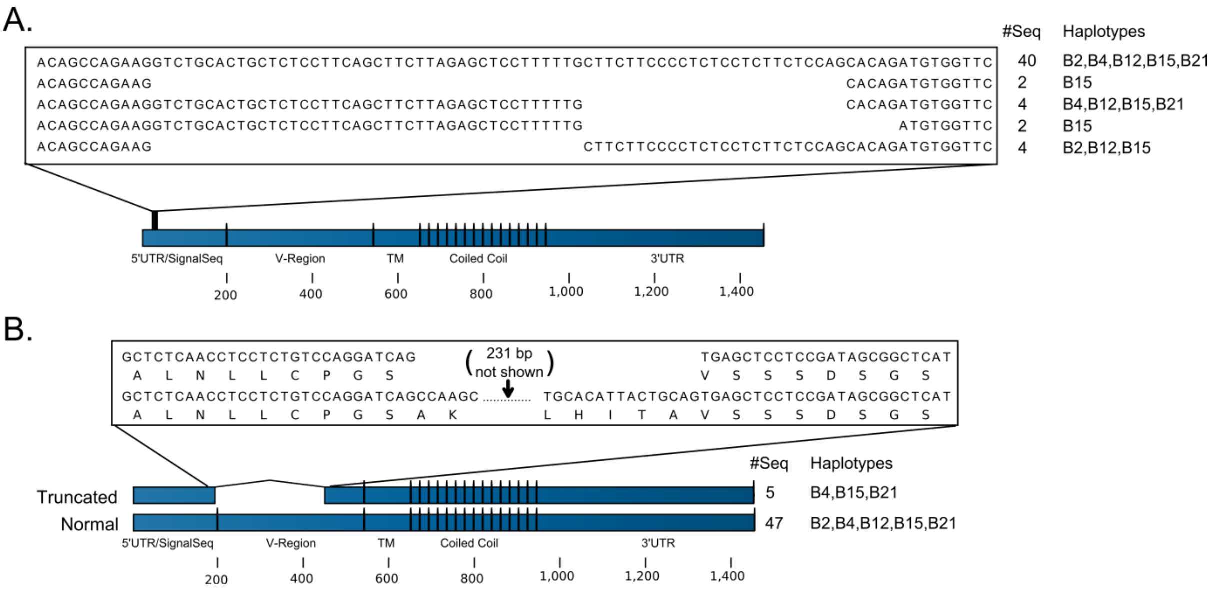

Figure S2. BG0 has indel variation (apparently alternative splicing) within the 5'UTR exon and the V domain exon. A. Roughly 10% of BG0 transcripts have alternative splicing in the 5'UTR ending at or near the start codon (ATG being positions 1087-1089 in Fig. S2 of Salomonsen et al 2014 PLoS Genet, reference 17; positions 83-85 on the top line in this figure), as shown in the cartoon and the sequences in the box (with the first 6 nucleotides being the end of the primer). The top sequence in the box is the genomic sequence. A canonical splice donor site removes introns starting at position 1016 (position 12 in this figure) and a non-canonical splice donor site removes introns starting at position 1057 (position 53). A non-canonical splice acceptor site removes introns ending at position 1056 (position 52 in this figure), and canonical splice acceptor sites remove introns ending at positions 1081 and 1086 (positions 77 and 82). The number of times each sequence was found in a particular chicken line is shown on the right of the figure. B. Roughly 10% of BG0 transcripts have a truncated V domain sequence, due to splicing from the end of the previous exon (ending with GGATCAG in this figure, followed by the canonical splice donor GT at positions 1384-1385 in Fig. S2 of Salomonsen et al 2014 PLoS Genet, reference 17) to a cryptic but canonical splice site 255 nucleotides into the exon (including upstream pyrimidine tract and ending in CAG, positions 1985-1987, with coding starting at position 1988 with TGAGC), as shown in the cartoon and the sequences in the box. The number of times each sequence was found in a particular chicken line is shown on the right of the figure. (Chattaway J et al. 2016. Open Biology)

Chattaway J, Ramirez-Valdez RA, Chappell PE, Caesar JJE, Lea SM and Kaufman J.
Different modes of variation for each BG lineage suggest different functions. 2016. Open Biology
Electronic Supplementary Material

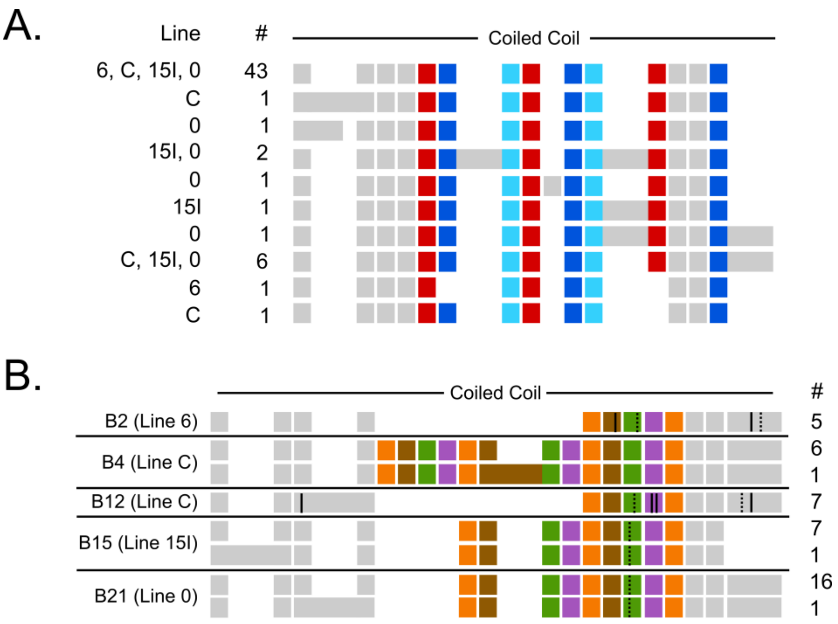

C.

	Lack extra codon	Contain extra codon
B2 (Line 6)	4	1
B4 (Line C)	6	1
B12 (Line C)	1	6
B15 (Line 15I)	8	0
B21 (Line 0)	8	9

Figure S3. For each line and haplotype, there is one predominant transcript for BG0 and for BG1, but some minor variants arise from differences in the cytoplasmic tail. Sequences represented as in Fig. 2. A. BG0 variants have intron read-through after the first cytoplasmic exon, in the three exon repeat or after the last coding exon, along with one transcript with an apparent extra exon within the three exon repeat. B. BG1 variants have intron read-through after the first cytoplasmic exon, after the third cytoplasmic exon, or in the four exon repeat. C. BG1 transcripts also have two splice site acceptors at the beginning of the first cytoplasmic exon, with the earlier acceptor site leading to an extra codon, giving the exon 24 amino acids rather than 21. These two sites appear to be used differentially in each haplotype, except in the B21 haplotype where they are used roughly at the same level. A similar phenomenon may be occurring at the start of the sequence corresponding to cytoplasmic exon 8 in BG1 from the B2 haplotype. (Chattaway J et al. 2016. Open Biology)

Chattaway J, Ramirez-Valdez RA, Chappell PE, Caesar JJE, Lea SM and Kaufman J.
Different modes of variation for each BG lineage suggest different functions. 2016. Open Biology
Electronic Supplementary Material

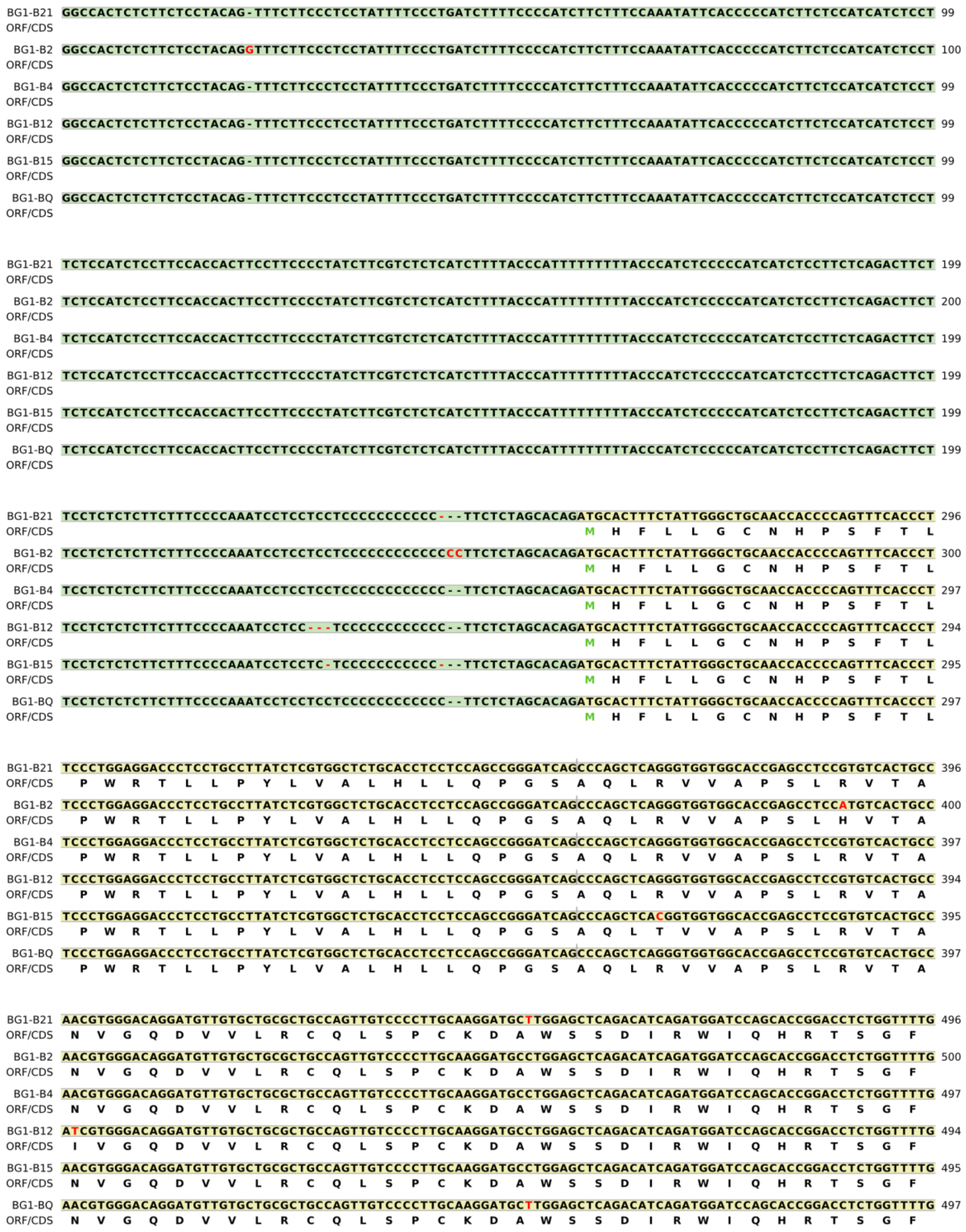

Fig. S4, continued next page.

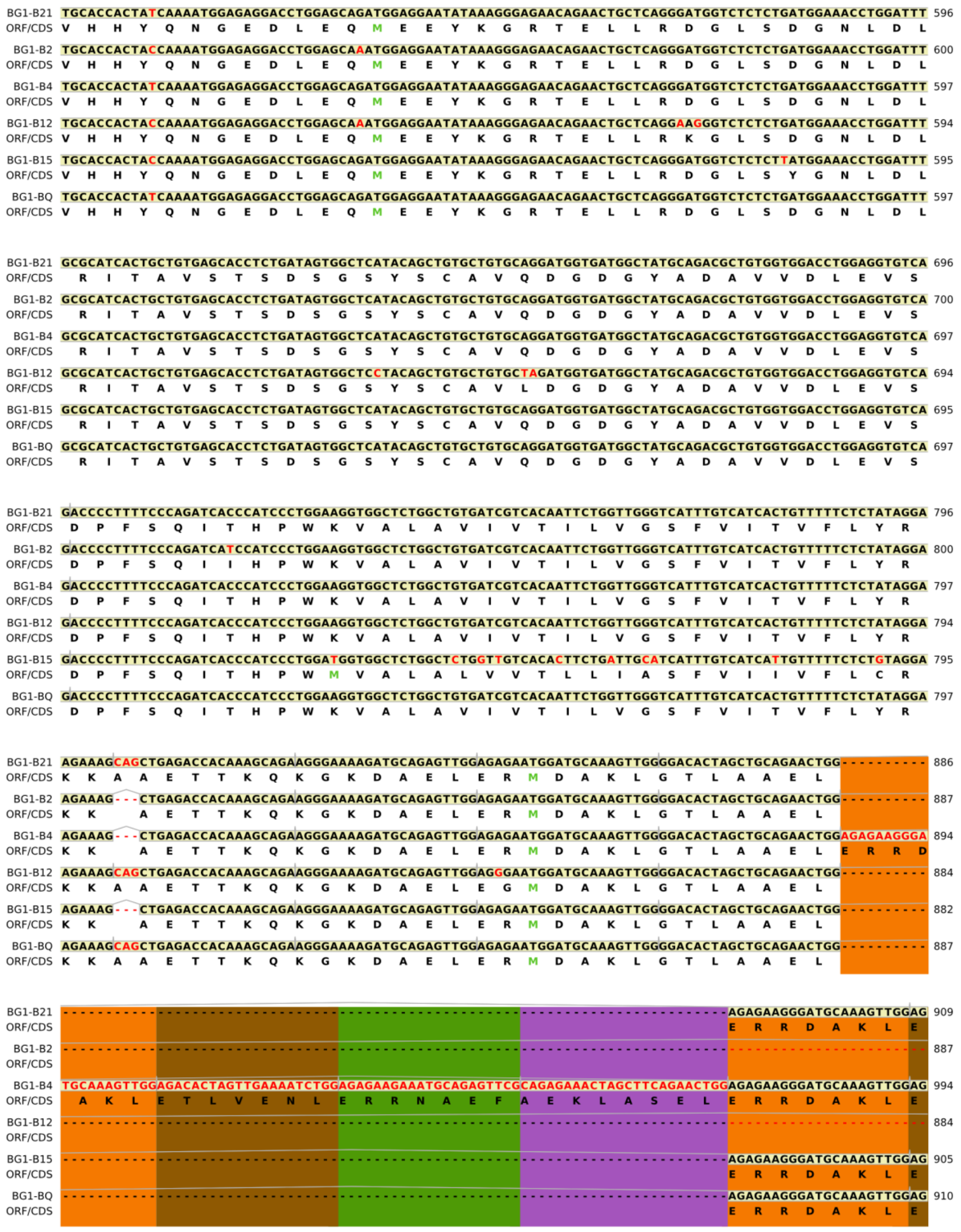

Fig. S4, continued next page.

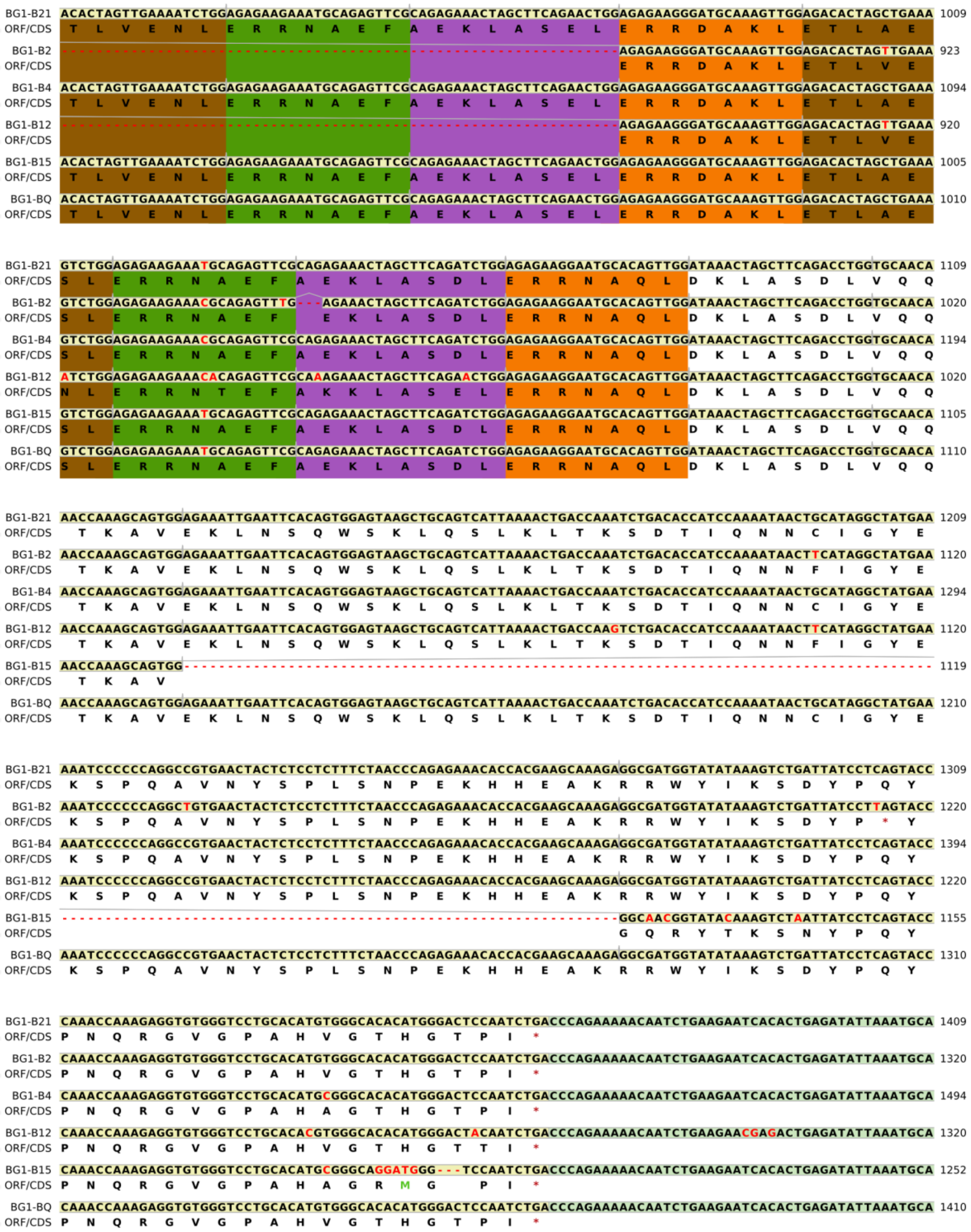

Fig. S4, continued next page.

```
BG1-B21 CCTCAGTTACCACTGGTGTTAAAAATCCATACTGGAAGAAGAGGAGACAGTGTTTGTATTGAGTGAGAACACTGCAGTTCTGTGAGCCAAAGCTGCCTGA 1509
ORF/CDS
 BG1-B2 CCTCAGTTACCACTGGTGTTAAAAATCCATACTGGAAGAAGAGGAGACAGTGTTTGTATTGAGTGAGAACACTGCAGTTCTGTGAGCCAAAGCTGCCTGA 1420
ORF/CDS
 BG1-B4 CCTCAGTTACCACTGGTGTTAAAAATCCATACTGGAAGAAGAGGAGACAGTGTTTGTATTGAGTGAGAACACTGCAGTTCTGTGAGCCAAAGCTGCCTGA 1594
ORF/CDS
BG1-B12 CCTCAGTTACCACTGGTGTTAAAAATCCATACTGGAAGAAGAGGAGACAGTGTTTGTATTGAGTGAGAACACTGCAGTTCTGTGAGCCAAAGCTGCCTGA 1420
ORF/CDS
BG1-B15 CCTCAGTTACCACTGGTGTTAAAAATCCATACTGGAAGAAGAGGAGACAGTGTTTGTATTGAGTGAGAACACTGCAGTTCTGTGAGCCAAAGCTGCCTGA 1352
ORF/CDS
 BG1-BQ CCTCAGTTACCACTGGTGTTAAAAATCCATACTGGAAGAAGAGGAGACAGTGTTTGTATTGAGTGAGAACACTGCAGTTCTGTGAGCCAAAGCTGCCTGA 1510
ORF/CDS

BG1-B21 GAAACCACCGAACTGAGGGTGTGTGACCTCCAACTCAAATCCAATTGGAAGAAAGAAACCACAGGAAGGAAGAAAATGGGTGGAAGACAGAGATCCTGGA 1609
ORF/CDS
 BG1-B2 GAAACCACCGAACTGAGGGTGTGTGACCTCCAACTCAAATCCAATTGGAAGAAAGAAACCACAGGAAGGAAGAAAATGGGTGGAAGACAGAGATCCTGGA 1520
ORF/CDS
 BG1-B4 GAAACCACCGAACTGAGGGTGTGTGACCTCCAACTCAAATCCAATTGGAAGAAAGAAACCACAGGAAGGAAGAAAATGGGTGGAAGACAGAGATCCTGGA 1694
ORF/CDS
BG1-B12 GAAACCACCGAACTGAGGGTGTGTGACCTCCAACTCAAATCCAATTGGAAGAAAGAAACCACAGGAAGGAAGAAAATGGGTGGAAGACAGAGATCCTGGA 1520
ORF/CDS
BG1-B15 GAAACCACCGAACTGAGGGTGTGTGACCTCCAACTCAAATCCAATTGGAAGAAAGAAACCACAGGAAGGAAGAAAATGGGTGGAAGACAGAGATCCTGGA 1452
ORF/CDS
 BG1-BQ GAAACCACCGAACTGAGGGTGTGTGACCTCCAACTCAAATCCAATTGGAAGAAAGAAACCACAGGAAGGAAGAAAATGGGTGGAAGACAGAGATCCTGGA 1610
ORF/CDS

BG1-B21 AAAGATATGGGCATTTTGGGAAACAGTGTGACCACGTATCAGGATTCATGGAAATCCAATG--------------------------------------- 1670
ORF/CDS
 BG1-B2 AAAGATATGGGCATTTTGGGGAACAGTGTGACCATGTATCAGGATTCATGGAAATCCAATGAATATGTGTAGTGGAATTCCAGGGTCACAGCCTGAACCA 1620
ORF/CDS
 BG1-B4 AAAGATATGGGCATTTTGGGGAACAGTGTGACCACGTATCAGGATTCATGGAAATCCAATG--------------------------------------- 1755
ORF/CDS
BG1-B12 AAAGATATGGGCATTTTGGGAAACAGTGTGACCACGTATCAGGATTCATGGAAATCCAATG--------------------------------------- 1581
ORF/CDS
BG1-B15 AAAGATATGGGCATTTTGGGAAACAGTGTGACCACGTATCAGGATTCATGGAAATCCAATG--------------------------------------- 1513
ORF/CDS
 BG1-BQ AAAGATATGGGCATTTTGGGAAACAGTGTGACCACGTATCAGGATTCATGGAAATCCAATG--------------------------------------- 1671
ORF/CDS

BG1-B21 ---------------------------------------------------------------------------------------------------- 1670
ORF/CDS
 BG1-B2 ATGGGTGGGCTCTGTGAGTGTGTGAGTGCGGCGGCGCGGCGGTACGAACGGGGGAGGGACACATTCCTCAGCTCGGCTCGGGCTGCGCGCTGCTACCGGG 1720
ORF/CDS
 BG1-B4 ---------------------------------------------------------------------------------------------------- 1755
ORF/CDS
BG1-B12 ---------------------------------------------------------------------------------------------------- 1581
ORF/CDS
BG1-B15 ---------------------------------------------------------------------------------------------------- 1513
ORF/CDS
 BG1-BQ ---------------------------------------------------------------------------------------------------- 1671
ORF/CDS

BG1-B21 --------------------------------------------------------------------------------------AATATGTAAGGCTT 1684
ORF/CDS
 BG1-B2 AGGGGTGAGTCGATTCTTTTTGATATCTTTCCCTCGTGTGCCTATTTCTTAAATAAAGGCTTTGTCATCACCTTCATTTGCCCTACAATATGTAAGGCTT 1820
ORF/CDS
 BG1-B4 --------------------------------------------------------------------------------------AATATGTAAGGCTT 1769
ORF/CDS
BG1-B12 --------------------------------------------------------------------------------------AATATGTAAGGCTT 1595
ORF/CDS
BG1-B15 --------------------------------------------------------------------------------------AATATGTAAGGCTT 1527
ORF/CDS
 BG1-BQ --------------------------------------------------------------------------------------AATATGTAAGGCTT 1685
ORF/CDS
```

Fig. S4, continued next page.

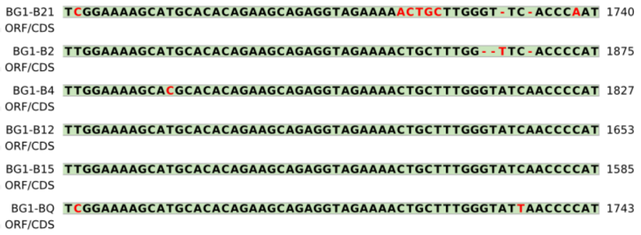

Figure S4. Nucleotide and protein sequence alignment of the consensus BG1 cDNAs from each haplotype. Position numbers are relative to the start of the 5' primer binding site, which covers up to positions 22 (with the variation at position 23 in BG1-B2 appearing to be an artefact, based on comparison with the genomic sequence). The 3’ primer binding site starts at position 1720 of the BG1-BQ sequence (meaning that the many variant positions found at the end of the sequences are likely to be artefacts). Consensus is defined as containing the exons for the 5'UTR, V domain, transmembrane region, all of the cytoplasmic tail, and the 3'UTR, but conditionally expressed indels and read-through exons are excluded. Open reading frames are highlighted in yellow, and protein sequence is displayed below the open reading frame. Exons are denoted by faint grey ticks above the coding sequence. Short cytoplasmic exons are coloured with the same colours as in Fig. 2. Stop codons are shown as asterisks, gaps are shown as dashes, and polymorphic residues are highlighted in red. There is no meaning to the fact that Met residues (M) are shown in green. Two sequences were found for B15, differing by the addition of a T between positions C241 and T242 in the sequence presented. These sequences (without the primer sites) were deposited in Genbank (with the following accession numbers): BG1B21 (KX686503), BG1B2 (KX686502), BG1B4 (KX686504), BG1B12 (KX686501) and BG1B15 (KX686505).

Comparisons of these cDNA sequences with available genomic sequences show some variation. Between the two B21 sequences (KX686503 and AB426152), there is 1 SNP in the 10th cytoplasmic exon (along with 1 other SNP at the end of the 3’UTR that could not be compared since the genomic sequence starts in the 3’UTR). Between the two B2 sequences (KX686502 and AB426141), there is 1 SNP and a 4 nucleotide insertion in the run of Cs in the 5’UTR, a 225 nucleotide insertion near the end of the 3’UTR, and 1 SNP in at the end of the 3’UTR (along with 2 other SNPs that could not be compared since the genomic sequence starts in the 3’UTR). Search of ENA database shows that some chicken MHC haplotypes (such as EM_OV:FJ770457 and EM_OV: FJ770458) do have the 225 nucleotide insertion, so it is likely to be a polymorphism. Between the two B12 sequences (KX686501 and AB426147), there are 2 SNPs in the run of Cs in the 5’UTR between the two B12 sequences (KX686501 and AB426147). Between the B4 cDNA sequence (KX686504) and the B13 genomic sequence (AB426148), there is no obvious alignment until the 5th cytoplasmic exon (which is the exon of the first repeat, coloured orange above); in fact this 5’ region of the B13 sequence fails to align with the other genomic sequences analysed. In the portion that can be compared, there is 1 SNP in 10th cytoplasmic exon and 1 SNP in the 3’UTR (along with 2 other SNPs that could not be compared since the genomic sequence starts in the 3’UTR). Between the two B15 sequences (KX686505 and AB426149), there is 1 SNP in the run of Cs in the 5’UTR, and 1 SNP each in the 8th and 10th cytoplasmic exons.

(Chattaway J et al. 2016. Open Biology)
